# Association of the ward pharmacy service with active implementation of therapeutic drug monitoring for vancomycin and teicoplanin—an epidemiological surveillance study using Japanese large health insurance claims database

**DOI:** 10.1186/s40780-020-00174-8

**Published:** 2020-08-18

**Authors:** Shungo Imai, Kenji Momo, Hitoshi Kashiwagi, Takayuki Miyai, Mitsuru Sugawara, Yoh Takekuma

**Affiliations:** 1 Faculty of Pharmaceutical Sciences, Hokkaido University, Kita 12-jo Nishi 6-chome, Kita-ku, Sapporo, 060-0812 Japan; 2 Department of Hospital Pharmaceutics, School of Pharmacy, Showa University, 1-5-8 Hatanodai, Shinagawa-ku, Tokyo, 142-8555 Japan; 3 Graduate School of Life Science, Hokkaido University, Kita 10-jo Nishi 8-chome, Kita-ku, Sapporo, 060-0810 Japan; 4 Department of Pharmacy, Hokkaido University Hospital, Kita 14-jo, Nishi 5-chome, Kita-ku, Sapporo, 060-8648 Japan

**Keywords:** Therapeutic drug monitoring, Ward pharmacists, Health insurance claims

## Abstract

**Background:**

Ward pharmacists are required for the active implementation of therapeutic drug monitoring (TDM). This epidemiological study verified whether Japanese ward pharmacists contribute to improving the TDM implementation proportions of anti-methicillin-resistant *Staphylococcus aureus* (MRSA) agents using the large health insurance claims database.

**Methods:**

The patients who received intravenous anti-MRSA agents from April 2012 to March 2017 were enrolled. We defined ward pharmacy service as the “drug management and guidance fee” and/or “inpatient pharmaceutical services premium”. In addition, implementation of TDM was identified by “the specific drug treatment management fee”. We compared the proportions of TDM implementation for vancomycin (VCM), teicoplanin (TEIC), and arbekacin (ABK) in the ward and non-ward pharmacy service groups. To avoid confounding, the propensity score method was employed. Moreover, the clinical variables affecting TDM implementation in each anti-MRSA agent were analyzed by using a multiple logistic regression model.

**Results:**

The following number of patients were included in the study: VCM (*n* = 2138), TEIC (*n* = 596), and ABK (*n* = 142). After propensity score matching, the proportions of TDM implementation for VCM and TEIC were higher in the ward pharmacy service group than in the non-ward pharmacy service group (VCM: 69.2% vs 60.3%, TEIC: 51.4% vs 34.7%), while no significant difference was observed for ABK (21.2% vs 23.1%). As independent clinical variables affecting TDM implementation for VCM and TEIC, several clinical variables, including ward pharmacy services, were extracted. In contrast, no clinical variables were extracted for ABK.

**Conclusions:**

We found that the ward pharmacy service is associated with the active implementation of TDM for anti-MRSA agents, such as VCM and TEIC.

## Background

Therapeutic drug monitoring (TDM) is a useful tool to improve the efficacy and safety of pharmacotherapy [1–4]. TDM is usually targeted for the drugs that fall in the narrow therapeutic range or are addictive. In Japan, there is a “specific drug treatment management fee”, a medical fee related to TDM, e.g. which covers anti-methicillin-resistant *Staphylococcus aureus* (MRSA) agents, antiepileptic agents, immunosuppressants, and arrhythmia agents [5]. Among them, anti-MRSA agents are widely subjected to TDM by pharmacists, and its usefulness has been previously reported [6–9]. For example, Komoto et al. reported that pharmacist intervention of dose settings of vancomycin (VCM) was associated with maintaining a balance between the efficacy and safety of VCM therapy [6]. Masuda et al. found that pharmacist intervention of TDM reduce the risk of VCM-induced nephrotoxicity [7]. In addition, Okada et al. validated the effectiveness of hematological ward pharmacist interventions of anti-MRSA agents, such as VCM and teicoplanin (TEIC) [8]. Further, they showed that the proportion of achieving the therapeutic range was significantly improved by pharmacist intervention [8]. Thus, these results strongly indicated that pharmacists could contribute to improving the efficacy and safety of anti-MRSA agents by TDM implementation.

In Japan, there are two medical fees associated with ward pharmacy services, “drug management and guidance fee” and “inpatient pharmaceutical services premium” [5]. The “drug management and guidance fee” can be calculated up to once a week for the inpatients by implementation of the patient compliance instruction and pharmaceutical management, such as evaluating the dosage, route of administration, dosing proportion, and drug-drug interactions. The “inpatient pharmaceutical services premium” is like a “hospital fee”. For calculating this, ward pharmacists are required to station at each ward for more than 20 h/week and perform pre-defined pharmaceutical services. For example, one of the requirements is the description of “the ward pharmacist should set the appropriate dosage before administration, especially for the drugs that need to be safely managed and require calculation of flow proportion or dosage” [5]. Thus, both of medical fee can be interpreted as requiring ward pharmacists to contribute to the active implementation of TDM.

As described above, the effectiveness of pharmacist interventions in TDM have been reported previously [6–9]. However, almost all these studies were performed in a single center. Therefore, nationwide verification is needed to determine whether Japanese ward pharmacists can contribute to improving the proportion of TDM implementation for anti-MRSA agents. Recently, medical “big data”, including claims databases, have been used for research purposes [10–12]. For example, the large Japanese health insurance claims database, named “JMDC claims database” was constructed by the JMDC Inc., Tokyo [13]. This database has medical and pharmacy claims, with a cumulative population of about 5.6 million (as of June 2018), which is about 5% of the population of Japan. Therefore, such a claims database can be used to conduct the abovementioned nationwide surveillance. However, the Japanese claims database includes only limited data; for example, it does not contain clinical data [13]. Thus, the implementation of ward pharmacy service and TDM would need to be detected based on calculation of the related medical fee.

Therefore, this study surveyed whether calculation of “drug management and guidance fee” and “inpatient pharmaceutical services premium” (i.e. implementation of ward pharmacy service) is related to the calculation of “specific drug treatment management fee” (i.e. implementation of TDM) using large health insurance claims database.

## Methods

### Data sources

As described above, the JMDC claims database was employed [13]. This database contains millions of patient records from more than 100 health-insurance unions. However, since it includes mainly records for employees from large companies and their families, information is limited to patients older than 65 years and does not contain data for patients older than 75 years. Information that can be obtained from this database includes encrypted personal identifiers, age, sex, patient’s diagnosis, medical services, drug prescriptions (including dose and number of prescription days), inpatient or outpatient status, the size of the medical facility, and clinical department. All drugs were coded using the Anatomical Therapeutic Chemical Classification (ATC) System.

### Study population and data collection

In this study, we investigated the proportions of TDM implementation of anti-MRSA agents, such as VCM, TEIC, and arbekacin (ABK). Patients who received intravenous injections of these anti-MRSA agents for more than three days from April 2012 to March 2017 during hospitalization were enrolled. VCM, TEIC, and ABK were identified using the ATC system, with the codes J01XA01, J01XA02, and J01GB12, respectively. The clinical departments and the institutions were identified by the text codes and the institutional IDs, respectively. For each anti-MRSA agent, if a patient received multiple administrations during the study period, only the first administration was included in the analysis.

The implementation of TDM was defined as calculating the “specific drug treatment management fee” during the administration period. In addition, implementation of ward pharmacy service was defined as the calculation of “drug management and guidance fee” and/ or “inpatient pharmaceutical services” during or within one week before or after the administration period. The data regarding age, sex (male/ female), duration of treatment with each anti-MRSA agent, number of hospital beds, and clinical departments that prescribed the anti-MRSA agent were collected. The duration of treatment was evaluated as the total number of prescription days. If there was a lapse of more than seven days in the administration of VCM or TEIC, or three days in the administration of ABK, it was considered the end of administration. For evaluating the clinical departments, the top five departments that prescribed each anti-MRSA agent frequently were extracted. The number of hospital beds was categorized as ≤199 beds, 200–499 beds, and ≥ 500 beds.

### Study endpoints

Eligible patients were divided into ward pharmacy service group and non-ward pharmacy service group. As the primary endpoint, the proportions of TDM implementation were compared for VCM, TEIC, and ABK after propensity score matching between the two groups [14]. In addition, we evaluated the proportion of TDM implementation in pediatric (under 18-years old) and non-pediatric patients (18 years of age and older) after propensity score matching.

As the secondary endpoint, clinical variables affecting TDM implementation for each anti-MRSA agent were analyzed after propensity score matching. For this analysis, the implementation of ward pharmacy service was evaluated in addition to the above- mentioned patient characteristics.

### Data analyses

To compare the proportions and to analyze the clinical variables affecting TDM implementation, we employed propensity score matching because the data were not randomized. Considering this, the factors that could potentially affect implementation of ward pharmacy service (age, sex, treatment duration, number of hospital beds, and clinical departments that prescribed the anti-MRSA agents) in a multivariate logistic model were included. As the reason for extracting these variables, for hospitals not calculating “inpatient pharmaceutical services premium”, age, sex, treatment duration, and clinical departments might potentially affect to perform the “drug management and guidance”. That is, some hospitals may perform the “drug management and guidance” by pharmacists for a limited number of wards and patients. Also, we selected the “number of hospital beds” as potential variables because small hospitals may have difficulty in performing ward pharmacy service.

The propensity score was calculated from the selected significant factors. Pairs of ward pharmacy service group and non-ward pharmacy service group were matched by nearest neighbor matching within a caliper (0.2 of the standard deviation of the logit of the propensity score) [15, 16]. After matching the propensity scores, the statistical balance between the two groups was evaluated. A standardized difference <  0.1 was considered an adequate variable value after propensity matching [17]. Then, we compared the proportions of TDM implementation for each anti-MRSA agent between the ward pharmacy service group and non-ward pharmacy service group. For comparison of the categorical variables, Pearson’s chi-square or Fisher’s exact test were employed. Fisher’s exact test was used if over 20% of the cells had expected frequency less than 5 [18]. All the continuous variables were compared using Mann-Whitney U test because they conformed to the non-normal distribution by Shapiro-Wilk test or Kolmogorov-Smirnov test.

The present study analyzed the association between some clinical variables (age, sex, treatment duration, number of hospital beds, and clinical departments that prescribed the anti-MRSA agents) and TDM implementation including ward pharmacy and non-ward pharmacy services (after propensity score matching) using multiple logistic regression. The justification for selecting these potential variables were: (1) age; blood collection from pediatric patients may present a challenge, (2) sex; prevalence of complications that affect the pharmacokinetics of VCM, TEIC and ABK are often different between men and women (e.g., chronic kidney disease [19]), (3) treatment duration; prolonged treatments are generally associated with increased occurrence of side effects [20, 21], (4) number of hospital beds; small hospitals may have difficulty in performing TDM [22, 23], (5) clinical departments; surgery departments may have a low proportion of TDM (e.g., perioperative-prophylactic antibiotics), (6) ward pharmacy service; ward pharmacists may contribute to the active implementation of TDM.

In all statistical analyses, *P* value of ≤0.05 was considered to be significantly different. We employed statistical analysis software JMP 14® (SAS Institute Inc., Cary, NC, USA).

## Results

### Comparison of proportions of TDM implementation

**Fig. 1:**
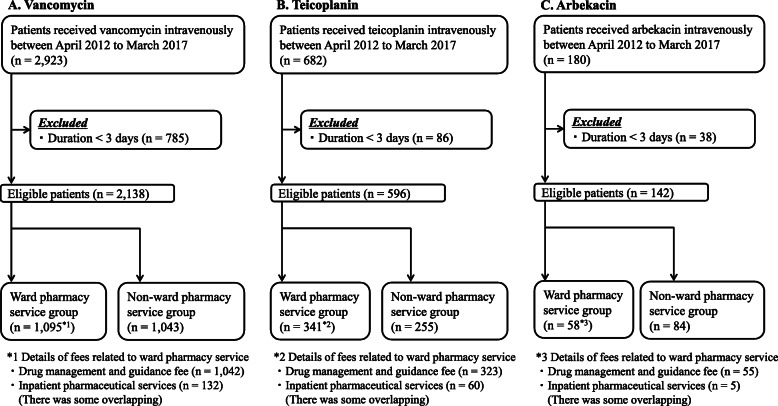
Flowchart of patients included in this study for each anti-MRSA agent. MRSA: methicillin-resistant *Staphylococcus aureus*

As shown in Fig. 1, the following number of patients were included in the study: VCM (*n* = 2138), TEIC (*n* = 596), and ABK (*n* = 142), and were divided into ward pharmacy service group and non-ward pharmacy service group.

Additional files 1, 2 and 3 (Tables S1-S3) show the characteristics of the patients before and after the propensity score matching. No significant differences were observed for any anti-MRSA agents between the ward pharmacy service group and the non-ward pharmacy service group upon propensity score matching. The standardized differences < 0.1 were obtained for all variables, except ABK. In case of ABK (Additional file 3: Table S3), data on the top six clinical departments were extracted because of the same number of patients in two variables.

**Fig. 2:**
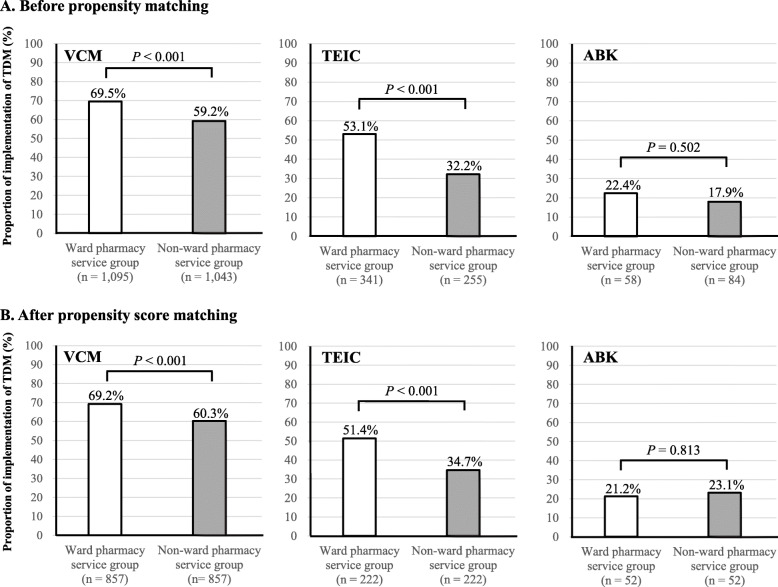
Comparison of the proportions of TDM implementation for each anti-MRSA agent before and after propensity score matching. Proportions of TDM implementation were compared by Chi-squared test. *P* value of ≤0.05 was considered statistically significant. TDM: therapeutic drug monitoring, VCM: vancomycin, TEIC: teicoplanin, ABK: arbekacin

Figure 2 shows the comparison of the proportions of TDM implementation for each anti-MRSA agent before and after propensity score matching. The proportion of TDM implementation for VCM and TEIC was significantly higher in the ward pharmacy service group than that in the non-ward pharmacy service group (VCM: 69.2 and 60.3%, TEIC: 51.4 and 34.7%, respectively, after propensity score matching). However, no significant difference was observed between the ward and non-ward pharmacy service groups for ABK (21.2 and 23.1%, respectively, after propensity score matching).

When pediatric (under 18-years old) and non-pediatric patients (18 years and older) were evaluated after propensity score matching (Table 1), higher proportions of TDM implementation were observed in the ward pharmacy service group, excluding pediatric patients treated with VCM as well as both pediatric and non-pediatric patients treated with ABK.

**Table 1:** Comparison of the proportions of TDM implementation for each anti-MRSA agent after propensity score matching when classified into pediatric patients (under 18-years old) and non-pediatric patients (18 years and older)

Description	Ward pharmacy service group	Non-ward pharmacy service group	*P*-value
Vancomycin			
Pediatric patients, n/n (%)	106/179 (59.2)	105/207 (50.7)	0.095 ^a)^
Non-pediatric patients, n/n (%)	487/678 (71.8)	412/650 (63.4)	0.001 ^a) *^
Teicoplanin			
Pediatric patients, n/n (%)	35/87 (40.2)	22/102 (21.6)	0.005 ^a) *^
Non-pediatric patients, n/n (%)	79/135 (58.5)	55/120 (45.8)	0.043 ^a) *^
Arbekacin			
Pediatric patients, n/n (%)	2/17 (11.8)	5/20 (25.0)	0.417 ^b)^
Non-pediatric patients, n/n (%)	9/35 (25.7)	7/32 (21.9)	0.713 ^a)^

*TDM* Therapeutic Drug Monitoring, *MRSA* methicillin-resistant *Staphylococcus aureus*. a) Chi-squared test, b) Fisher’s exact test. *P values ≤0.05 were considered statistically significant

### Clinical variables affecting TDM implementation

The clinical variables affecting TDM implementation in each anti-MRSA agent after propensity score matching are shown in Tables 2, 3 and 4.

**Table 2:** Clinical variables affecting TDM implementation of vancomycin

Characteristic	Univariate analysis			Multivariate analysis		
	OR	95% CI	*P* value	OR	95% CI	*P* value
Age (years)	1.005	1.001–1.009	0.024 ^*^	1.006	1.001–1.011	0.017 ^*^
Sex (male)	1.290	1.049–1.586	0.016 ^*^	1.221	0.984–1.515	0.069
Duration of vancomycin treatment (days)	1.056	1.038–1.074	< 0.001 ^*^	1.059	1.041–1.078	< 0.001 ^*^
Number of hospital beds						
≤ 199 beds	0.358	0.245–0.524	< 0.001 ^*^	0.299	0.197–0.452	< 0.001 ^*^
200–499 beds	1.103	0.875–1.390	0.408	1.079	0.831–1.399	0.569
≥ 500 beds	1.269	1.028–1.567	0.027 ^*^	–	–	–
Clinical departments of prescription of vancomycin						
Internal Medicine	1.099	0.899–1.343	0.357	1.162	0.917–1.472	0.214
Respiratory Medicine	1.436	0.937–2.200	0.097	1.473	0.925–2.344	0.103
Pediatrics	0.513	0.302–0.871	0.013 ^*^	0.680	0.365–1.267	0.224
Other internal medicine	2.568	1.287–5.126	0.007 ^*^	2.854	1.385–5.881	0.004 ^*^
Cardiology	0.697	0.258–1.881	0.476	1.090	0.379–3.133	0.873
Other departments	0.812	0.653–1.011	0.062	–	–	–
Ward pharmacy service	1.477	1.210–1.803	< 0.001 ^*^	1.514	1.232–1.861	< 0.001 ^*^

*TDM* Therapeutic Drug Monitoring, *OR* odds ratio, 95% CI: 95% confidence interval. *P-values ≤0.05 were considered statistically significant. The odds ratios of “number of hospital beds ≥500 beds” and “clinical departments of other departments” were not calculated because they had linear dependence with other factors

**Table 3:** Clinical variables affecting TDM implementation of teicoplanin

Characteristic	Univariate analysis			Multivariate analysis		
	OR	95% CI	*P* value	OR	95% CI	*P* value
Age (years)	1.015	1.007–1.022	< 0.001 ^*^	1.013	1.004–1.022	0.005 ^*^
Sex (male)	1.347	0.915–1.984	0.131	1.162	0.753–1.792	0.497
Duration of teicoplanin treatment (days)	1.061	1.033–1.091	< 0.001 ^*^	1.056	1.025–1.087	< 0.001 ^*^
Number of hospital beds						
≤ 199 beds	0.339	0.111–1.040	0.058	0.197	0.060–0.648	0.008 ^*^
200–499 beds	0.920	0.536–1.580	0.762	0.540	0.289–1.011	0.054
≥ 500 beds	1.388	0.846–2.277	0.194	–	–	–
Clinical departments of prescription of teicoplanin						
Internal Medicine	1.665	1.139–2.433	0.008 ^*^	2.560	1.590–4.121	< 0.001 ^*^
Respiratory Medicine	1.966	1.039–3.720	0.038 ^*^	3.049	1.433–6.490	0.004 ^*^
Gastroenterology	3.439	1.062–11.139	0.039 ^*^	3.272	0.899–11.903	0.072
Other departments	0.354	0.233–0.539	< 0.001 ^*^	–	–	–
Ward pharmacy service	1.988	1.357–2.911	< 0.001 ^*^	2.145	1.415–3.253	< 0.001 ^*^

*TDM* Therapeutic Drug Monitoring, *OR* odds ratio, 95% CI: 95% confidence interval. *P-values ≤0.05 were considered statistically significant. The odds ratios of “number of hospital beds ≥500 beds” and “clinical departments of other departments” were not calculated because they had linear dependence with other factors

**Table 4:** Clinical variables affecting TDM implementation of arbekacin

Characteristic	Univariate analysis			Multivariate analysis		
	OR	95% CI	*P* value	OR	95% CI	*P* value
Age (years)	1.003	0.985–1.020	0.767	1.006	0.988–1.025	0.504
Sex (male)	1.308	0.509–3.364	0.577	1.222	0.451–3.310	0.693
Duration of arbekacin treatment (days)	1.052	0.970–1.140	0.218	1.073	0.983–1.171	0.121
Number of hospital beds						
≤ 199 beds	0.606	0.124–2.953	0.535	0.326	0.051–2.083	0.236
200–499 beds	0.706	0.250–1.995	0.511	0.615	0.188–2.009	0.421
≥ 500 beds	1.657	0.633–4.339	0.304	–	–	–
Clinical departments of prescription of arbekacin						
Internal medicine	0.940	0.369–2.396	0.898	1.640	0.462–5.814	0.444
Respiratory medicine	2.632	0.674–10.270	0.164	3.158	0.608–16.403	0.171
Gastroenterology	0.691	0.077–6.228	0.742	0.614	0.046–8.158	0.712
Other departments	0.794	0.262–2.404	0.683	–	–	–
Ward pharmacy service	0.894	0.354–2.260	0.813	0.803	0.302–2.137	0.661

*TDM* Therapeutic Drug Monitoring, *OR* odds ratio, 95% CI: 95% confidence interval. The odds ratios of “number of hospital beds ≥500 beds” and “clinical departments of other departments” were not calculated because they had linear dependence with other factors

As shown in Table 2, age, duration of VCM treatment, number of hospital beds ≤199, clinical departments of other internal medicine, and ward pharmacy services were extracted as independent clinical variables for TDM implementation of VCM.

With regard to the patients who received TEIC treatment, the Pediatrics and Cardiovascular surgery clinical departments were excluded from extracting independent clinical variables because there were too few patients to analyze. As a result, age, duration of TEIC treatment, number of hospital beds ≤199, the clinical departments of Internal Medicine and Respiratory Medicine, and ward pharmacy services were extracted as independent clinical variables for TDM implementation of TEIC (Table 3).

In the patients who received ABK treatment, clinical departments of other internal medicine, Obstetrics and gynecology, and Pediatrics were excluded for the same reason as above. No independent clinical variables affecting the TDM implementation for ABK were extracted (Table 4).

## Discussion

In this study, we verified whether the ward pharmacists contribute to the active implementation of TDM in anti-MRSA agents using large health insurance claims database. The strength of this study was the provision of more generalized information compared with previous single-center studies [6–9].

To account for the confounding, we employed the propensity score method for comparison of the proportions of TDM implementation between the ward and non-ward pharmacy services [14]. As shown in Additional files 1, 2 and 3 (Tables S1-S3), the characteristics of the patients were observed to be significantly different before the propensity score matching. For example, age, treatment duration, number of hospital beds ≤199, and clinical departments (Internal Medicine, Pediatrics, and Cardiology) differed between the two groups for VCM (Additional file 1: Table S1). These results are reflective of the characteristics of the facility in which the ward pharmacy services can be performed. For instance, our results suggest that small hospitals may have difficulty in performing the ward pharmacy services, consistent with the previous surveillance reports [22, 23]. In addition, patients in the non-ward pharmacy service group for each drug before propensity score matching, especially TEIC and ABK, were significantly younger than those in the ward pharmacy service group. This was because there were many pediatric patients in the non-ward pharmacy service group. After propensity score matching, there was no significant difference between the two groups for any of the anti-MRSA agents. In addition, adequate variable balances were obtained for VCM and TEIC with standardized difference <  0.1 [17]. However, in case of ABK (Additional file 3: Table S3), the standardized differences of Internal Medicine and Gastroenterology clinical departments were > 0.1, suggesting imbalance in the corresponding baseline characteristics.

As shown in Fig. 2, the proportion of TDM implementation of VCM and TEIC was significantly higher in the ward pharmacy service group than the non-ward pharmacy service group (VCM: 69.2% vs 60.3%, TEIC: 51.4% vs 34.7%, after propensity score matching). Thus, these results suggest that the ward pharmacists contribute to the active implementation of TDM in VCM and TEIC, especially in case of TEIC, where a difference of 16.7% was observed between the two groups, suggesting great contribution by the ward pharmacists. However, ABK had the lowest proportion among the three anti-MRSA agents, and no difference was observed between the two groups. Although there was an imbalance in the baseline characteristics, these results indicate the ward pharmacists need to contribute to the active TDM implementation of ABK. Similar results were obtained when pediatric and non-pediatric patients were evaluated, excluding pediatric patients treated with VCM (Table 1).

As shown in Tables 2 and 3, age, duration of treatment, number of hospital beds ≤199, several clinical departments, and ward pharmacy services were extracted as the independent clinical variables of TDM implementation for VCM and TEIC. Extracting the ward pharmacy service also indicated the contribution of the ward pharmacists to the active TDM implementation of VCM and TEIC. The patient’s age showed higher odds ratio (OR) because performing the blood collection on the pediatric patients might have been challenging. This result was consistent with Table 1, i.e. TDM was implemented in a higher proportion of non-pediatric patients than in pediatric patients with or without ward pharmacy service for VCM and TEIC. Furthermore, guidelines have described that TDM should be performed when the duration of treatment is over 3–5 days for VCM and over 4 days for TEIC [1, 24]; thus our result of “long-term duration of treatment was associated with TDM implementation” was reasonable. In addition, data extracted on the number of hospital beds ≤199 suggest that small hospitals had difficulty in performing TDM, which is consistent with previous reports [22, 23]. With regard to clinical departments, other internal medicine was extracted for VCM and Internal Medicine and Respiratory Medicine were extracted for TEIC. However, the details of the clinical departments, especially other internal medicine, were unknown because clinical departments could be identified based only on pre-categorized criteria in the claims database. Meanwhile, no independent clinical variables affecting TDM implementation for ABK were extracted (Table 4). This may be, in part, because we could not obtain data on enough patients for multivariate logistic regression analysis [25].

This study has several limitations. First, the implementation of TDM and ward pharmacy service were defined as the calculation of related medical fees. However, their accuracies have not been validated. In particular, while the implementation of TDM was identified by “the specific drug treatment management fee,” the calculation may have included for other drugs besides anti-MRSA agent. Second, we collected information about the duration of treatment based on the total number of prescription days, but we had no data regarding the actual administrations. Third, as JMDC claims database does not include clinical data, several factors could not be evaluated. Thus, our results may be based on factors other than the presence of ward pharmacists. Fourth, the JMDC claims database includes mainly company employees and their families, who are under 75 years of age. Therefore, this database included relatively high number of pediatric patients. In fact, the implementation proportions of TDM were lower than those of the previous study, i.e. VCM (99%), TEIC (97%), and ABK (93%) [23]. Although, simple comparison was difficult because this report surveyed the implementation proportions in the hospitals that performed TDM and did not evaluate the proportions for individual patients [23]. In addition, patients who were employed in small- and medium-sized companies and self-employed were not included in JMDC claims database. Therefore, the generalizability to other populations remain unclear. Fifth, we did not evaluate the wards where the patients were hospitalized. For example, the calculation methods for determining “inpatient pharmaceutical services premium” used by psychiatric wards and intensive-care units are different from those used by general wards [5].

Considering these limitations, we could not evaluate an overall “contribution” of ward pharmacy service to the implementation of TDM but were able to find a beneficial “association” in the case of VCM and TEIC. Importantly, this study is the first epidemiological verification of an association between ward pharmacy service and TDM implementation using a claims database. An additional novelty of our study is that it showed the usefulness of a large claims database to verify the efficacy of pharmacist intervention. Thus, our approach can be applied to other pharmacist interventions and can be developed further.

## Conclusions

We found that the ward pharmacy service is associated with the active implementation of TDM for anti-MRSA agents, such as VCM and TEIC.

## Supplementary information

**Additional file 1: Table S1.** Comparison of patient characteristics of vancomycin before and after propensity score matching. A standardized difference (Std diff) < 0.1 is generally accepted as an adequate variable balance after propensity matching, a) Mann-Whitney U test, b) Chi-squared test. *P values ≤0.05 were considered statistically significant.

**Additional file 2: Table S2.** Comparison of patient characteristics of teicoplanin before and after propensity score matching. A standardized difference (Std diff) < 0.1 is generally accepted as an adequate variable balance after propensity matching, a) Mann-Whitney U test, b) Chi-squared test, c) Fisher’s exact test. *P values ≤0.05 were considered statistically significant.

**Additional file 3: Table S3.** Comparison of patient characteristics of arbekacin before and after propensity score matching. A standardized difference (Std diff) < 0.1 is generally accepted as an adequate variable balance after propensity matching, a) Mann–Whitney U test, b) Chi-squared test, c) Fisher’s exact test. *P values ≤0.05 were considered statistically significant.

## Data Availability

All data generated or analyzed in this study are included within the article.

## Abbreviations

TDM: Therapeutic drug monitoring
MRSA: Methicillin-resistant *Staphylococcus aureus*
VCM: Vancomycin
TEIC: Teicoplanin
ABK: Arbekacin
ATC: Anatomical therapeutic chemical classification
OR: Odds ratio
