# Supplementary material for: Association of the ward pharmacy service with active implementation of therapeutic drug monitoring for vancomycin and teicoplanin—an epidemiological surveillance study using Japanese large health insurance claims database
Source: J Pharm Health Care Sci. 2020 Aug 18;6:18. doi: 10.1186/s40780-020-00174-8 (PMC7436959; doi:10.1186/s40780-020-00174-8)
Supplement: Supplementary file 1 — Additional file 1: Table S1. Comparison of patient characteristics of vancomycin before and after propensity score matching. A standardized difference (Std diff) < 0.1 is generally accepted as an adequate variable balance after propensity matching, a) Mann-Whitney U test, b) Chi-squared test. *P values ≤0.05 were considered statistically significant. [file 40780_2020_174_MOESM1_ESM.docx]

| Description | Before propensity matching | | | | After propensity score matching | | | |
| --- | --- | --- | --- | --- | --- | --- | --- | --- |
|  | Ward pharmacy service group (n=1,095) | Non-ward pharmacy service group (n=1,043) | *P*-value | Std diff | Ward pharmacy service group (n=857) | Non-ward pharmacy service group (n=857) | *P*-value | Std diff |
| Age (years), median (range) | 50 (0-74) | 41 (0-74) | < 0.001 ^a) *^ | 0.375 | 46 (0-74) | 47 (0-74) | 0.383 ^a)^ | 0.005 |
| Sex (male), n (%) | 724 (66.1) | 667 (64.0) | 0.297 ^b)^ | 0.045 | 557 (65.0) | 566 (66.0) | 0.647 ^b)^ | 0.022 |
| Sex (female), n (%) | 371 (33.9) | 376 (36.0) |  |  | 300 (35.0) | 291 (34.0) |  |  |
| Duration of vancomycin treatment (days), median (range) | 8 (3-141) | 7 (3-71) | < 0.001 ^b) *^ | 0.124 | 8 (3-84) | 8 (3-71) | 0.707 ^b)^ | 0.027 |
| Number of hospital beds, n (%) |  |  |  |  |  |  |  |  |
| ≤ 199 beds | 62 (5.66) | 82 (7.86) | 0.043 ^b)^ ^*^ | 0.088 | 60 (7.00) | 58 (6.77) | 0.849 ^b)^ | 0.009 |
| 200-499 beds | 300 (27.4) | 276 (26.5) | 0.626 ^b)^ | 0.021 | 209 (24.4) | 214 (25.0) | 0.779 ^b)^ | 0.014 |
| ≥ 500 beds | 733 (66.9) | 685 (65.7) | 0.536 ^b)^ | 0.027 | 588 (68.6) | 585 (68.3) | 0.876 ^b)^ | 0.008 |
| Clinical departments for prescription of vancomycin, n (%) |  |  |  |  |  |  |  |  |
| Internal Medicine | 680 (62.1) | 545 (52.3) | < 0.001 ^b) *^ | 0.200 | 503 (58.7) | 493 (57.5) | 0.624 ^b)^ | 0.024 |
| Respiratory Medicine | 68 (6.21) | 61 (5.85) | 0.726 ^b)^ | 0.015 | 53 (6.18) | 58 (6.77) | 0.624 ^b)^ | 0.024 |
| Pediatrics | 28 (2.56) | 73 (7.00) | < 0.001 ^b) *^ | 0.209 | 28 (3.27) | 29 (3.38) | 0.893 ^b)^ | 0.007 |
| Other internal medicine | 29 (2.65) | 39 (3.74) | 0.151 ^b)^ | 0.062 | 29 (3.38) | 27 (3.15) | 0.786 ^b)^ | 0.013 |
| Cardiology | 8 (0.73) | 26 (2.49) | < 0.001 ^b) *^ | 0.140 | 8 (0.93) | 8 (0.93) | 1.000 ^b)^ | 0.000 |
| Other departments | 282 (25.8) | 299 (28.7) | 0.130 ^b)^ | 0.066 | 236 (27.5) | 242 (28.2) | 0.747 ^b)^ | 0.016 |
