# Supplementary material for: Association of the ward pharmacy service with active implementation of therapeutic drug monitoring for vancomycin and teicoplanin—an epidemiological surveillance study using Japanese large health insurance claims database
Source: J Pharm Health Care Sci. 2020 Aug 18;6:18. doi: 10.1186/s40780-020-00174-8 (PMC7436959; doi:10.1186/s40780-020-00174-8)
Supplement: Supplementary file 2 — Additional file 2: Table S2. Comparison of patient characteristics of teicoplanin before and after propensity score matching. A standardized difference (Std diff) < 0.1 is generally accepted as an adequate variable balance after propensity matching, a) Mann-Whitney U test, b) Chi-squared test, c) Fisher’s exact test. *P values ≤0.05 were considered statistically significant. [file 40780_2020_174_MOESM2_ESM.docx]

| Description | Before propensity matching | | | | After propensity score matching | | | |
| --- | --- | --- | --- | --- | --- | --- | --- | --- |
|  | Ward pharmacy service group (n=341) | Non-ward pharmacy service group (n=255) | *P*-value | Std diff | Ward pharmacy service group (n=222) | Non-ward pharmacy service group (n=222) | *P*-value | Std diff |
| Age (years), median (range) | 40 (0-74) | 17 (0-74) | 0.002 ^a) *^ | 0.302 | 33 (0-74) | 25.5 (0-74) | 0.446 ^a)^ | 0.079 |
| Sex (male), n (%) | 226 (66.3) | 157 (61.6) | 0.236 ^b)^ | 0.098 | 129 (58.1) | 139 (62.6) | 0.332 ^b)^ | 0.092 |
| Sex (female), n (%) | 115 (33.7) | 98 (38.4) |  |  | 93 (41.9) | 83 (37.4) |  |  |
| Duration of teicoplanin treatment (days), median (range) | 9 (3-70) | 8 (3-80) | 0.077 ^b)^ | 0.032 | 9 (3-50) | 8 (3-52) | 0.347 ^b)^ | 0.025 |
| Number of hospital beds, n (%) |  |  |  |  |  |  |  |  |
| ≤ 199 beds | 9 (2.64) | 15 (5.88) | 0.046 ^b)^ | 0.161 | 9 (4.05) | 10 (4.50) | 0.815 ^b)^ | 0.022 |
| 200-499 beds | 61 (17.9) | 49 (19.2) | 0.680 ^b)^ | 0.034 | 31 (14.0) | 32 (14.4) | 0.892 ^b)^ | 0.013 |
| ≥ 500 beds | 271 (79.5) | 191 (74.9) | 0.186 ^b)^ | 0.109 | 182 (82.0) | 180 (81.1) | 0.807 ^b)^ | 0.023 |
| Clinical departments for prescription of teicoplanin, n (%) |  |  |  |  |  |  |  |  |
| Internal Medicine | 194 (56.9) | 117 (45.9) | 0.008 ^b) *^ | 0.222 | 111 (50.0) | 115 (51.8) | 0.704 ^b)^ | 0.036 |
| Respiratory Medicine | 25 (7.33) | 23 (9.02) | 0.454 ^b)^ | 0.062 | 23 (10.4) | 20 (9.01) | 0.630 ^b)^ | 0.046 |
| Pediatrics | 1 (0.29) | 19 (7.45) | < 0.001 ^b) *^ | 0.378 | 1 (0.45) | 1 (0.45) | 1.000 ^c)^ | 0.000 |
| Gastroenterology | 8 (2.35) | 8 (3.14) | 0.554 ^b)^ | 0.048 | 8 (3.60) | 6 (2.70) | 0.587 ^b)^ | 0.052 |
| Cardiovascular surgery | 8 (2.35) | 1 (0.39) | 0.086 ^c)^ | 0.169 | 1 (0.45) | 1 (0.45) | 1.000 ^c)^ | 0.000 |
| Other departments | 105 (30.8) | 87 (34.1) | 0.390 ^b)^ | 0.071 | 78 (35.1) | 79 (35.6) | 0.921 ^b)^ | 0.009 |
