# Supplementary material for: Association of the ward pharmacy service with active implementation of therapeutic drug monitoring for vancomycin and teicoplanin—an epidemiological surveillance study using Japanese large health insurance claims database
Source: J Pharm Health Care Sci. 2020 Aug 18;6:18. doi: 10.1186/s40780-020-00174-8 (PMC7436959; doi:10.1186/s40780-020-00174-8)
Supplement: Supplementary file 3 — Additional file 3: Table S3. Comparison of patient characteristics of arbekacin before and after propensity score matching. A standardized difference (Std diff) < 0.1 is generally accepted as an adequate variable balance after propensity matching, a) Mann–Whitney U test, b) Chi-squared test, c) Fisher’s exact test. *P values ≤0.05 were considered statistically significant. [file 40780_2020_174_MOESM3_ESM.docx]

| Description | Before propensity matching | | | | After propensity score matching | | | |
| --- | --- | --- | --- | --- | --- | --- | --- | --- |
|  | Ward pharmacy service group (n=58) | Non-ward pharmacy service group (n=84) | *P*-value | Std diff | Ward pharmacy service group (n=52) | Non-ward pharmacy service group (n=52) | *P*-value | Std diff |
| Age (years), median (range) | 48 (0-73) | 5.5 (0-73) | 0.001 ^a) *^ | 0.589 | 43.5 (0-73) | 40.5 (0-73) | 0.739 ^a)^ | 0.085 |
| Sex (male), n (%) | 32 (55.2) | 41 (48.8) | 0.456 ^b)^ | 0.128 | 29 (55.8) | 29 (55.8) | 1.000 ^b)^ | 0.000 |
| Sex (female), n (%) | 26 (44.8) | 43 (51.2) |  |  | 23 (44.2) | 23 (44.2) |  |  |
| Durations of arbekacin (days), median (range) | 7 (3-44) | 6 (3-26) | 0.154 ^b)^ | 0.246 | 7 (3-33) | 6.5 (3-26) | 0.736 ^b)^ | 0.007 |
| Number of hospital beds, n (%) |  |  |  |  |  |  |  |  |
| ≤ 199 beds | 7 (12.1) | 11 (13.1) | 0.857 ^b)^ | 0.031 | 7 (13.5) | 6 (11.5) | 0.767 ^b)^ | 0.058 |
| 200-499 beds | 16 (27.6) | 23 (27.4) | 0.979 ^b)^ | 0.005 | 16 (30.8) | 17 (32.7) | 0.833 ^b)^ | 0.041 |
| ≥ 500 beds | 35 (60.3) | 50 (59.5) | 0.922 ^b)^ | 0.017 | 29 (55.8) | 29 (55.8) | 1.000 ^b)^ | 0.000 |
| Clinical departments for prescription of arbekacin, n (%) |  |  |  |  |  |  |  |  |
| Internal medicine | 33 (56.9) | 42 (50.0) | 0.418 ^b)^ | 0.139 | 32 (61.5) | 28 (53.8) | 0.427 ^b)^ | 0.156 |
| Respiratory medicine | 5 (8.62) | 9 (10.7) | 0.681 ^b)^ | 0.071 | 5 (9.62) | 5 (9.62) | 1.000 ^b)^ | 0.000 |
| Gastroenterology | 3 (5.17) | 5 (5.95) | 1.000 ^c)^ | 0.034 | 2 (3.85) | 4 (7.69) | 0.678 ^c)^ | 0.166 |
| Other internal medicine | 1 (1.72) | 2 (2.38) | 1.000 ^c)^ | 0.046 | 1 (1.92) | 1 (1.92) | 1.000 ^c)^ | 0.000 |
| Obstetrics and gynecology | 0 (0.0) | 3 (3.57) | 0.270 ^c)^ | 0.272 | 0 (0.0) | 0 (0.0) | － | － |
| Pediatrics | 0 (0.0) | 3 (3.57) | 0.270 ^c)^ | 0.272 | 0 (0.0) | 0 (0.0) | － | － |
| Other departments | 16 (27.6) | 20 (23.8) | 0.6111 ^b)^ | 0.087 | 12 (23.1) | 14 (26.9) | 0.651 ^b)^ | 0.089 |
